# Supplementary figures and images for: IsRNAcirc: 3D structure prediction of circular RNAs based on coarse-grained molecular dynamics simulation
Source: PLoS Comput Biol. 2024 Oct 28;20(10):e1012293. doi: 10.1371/journal.pcbi.1012293 (PMC11542809; doi:10.1371/journal.pcbi.1012293)

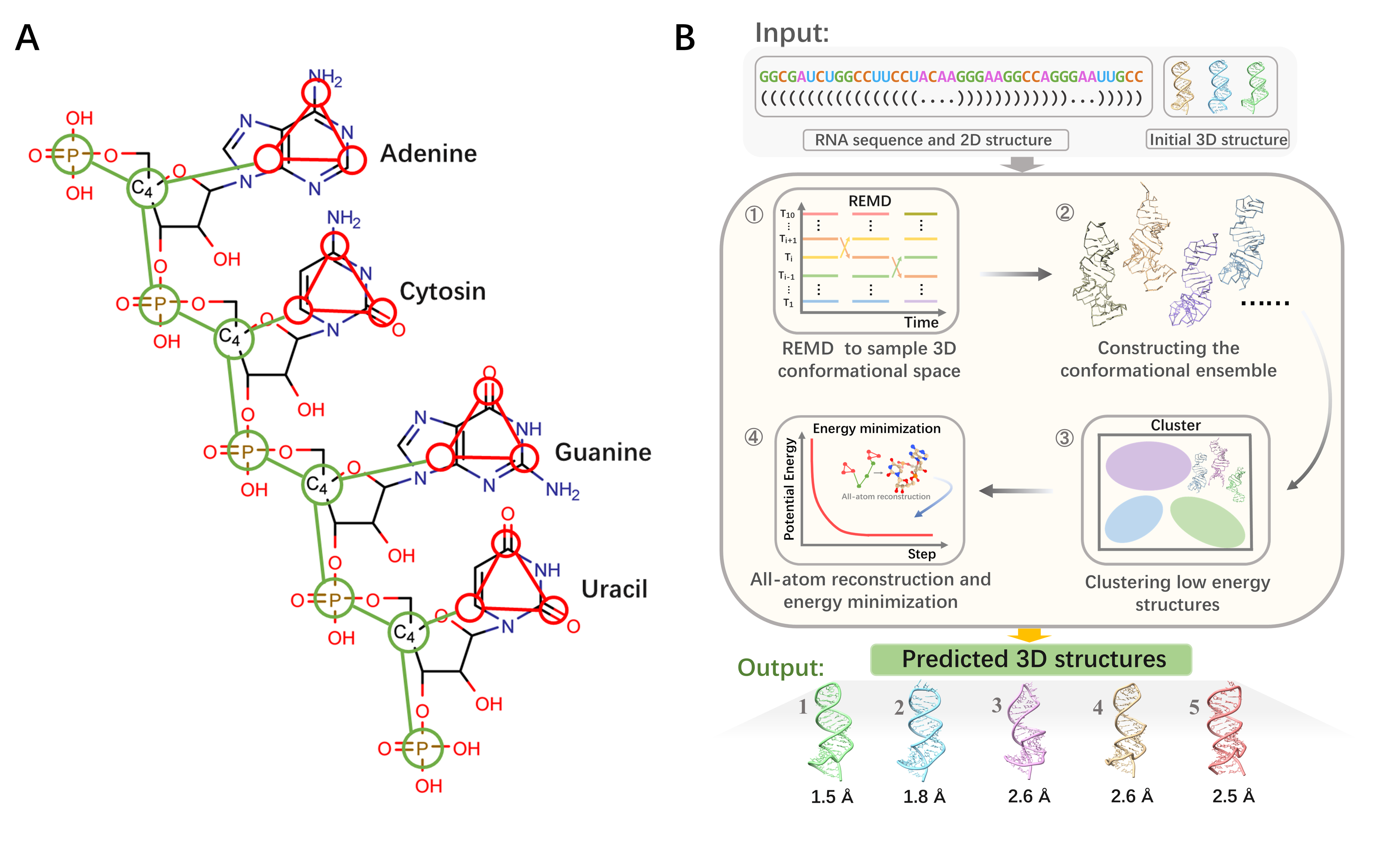

Supplement: S1 Fig — (A) Mapping relationships between the all-atom model and CG representation in IsRNA2: the backbone (colored by green) is represented by two CG beads (bead P located on the atom P for the phosphate group and bead S located on the atom C4’ for the ribose ring) and the nucleobase (colored by pink) is represented by three CG beads (each bead located at the center of mass of the related heavy-atom group). (B) An illustrated example to display the workflow for linear RNA 3D structure prediction by IsRNA2 model. Using sequence, 2D structure, and initial 3D structure as input, IsRNA2 employs a four-step process (conformation sampling through REMD, conformational ensemble construction, clustering procedure, and all-atom reconstruction and energy minimization) to generate the predicted 3D structures. For reference, the RMSD of each predicted model relative to the native structure (PDB ID: 1Z5C) was also shown. (TIF) [file pcbi.1012293.s001.tif]

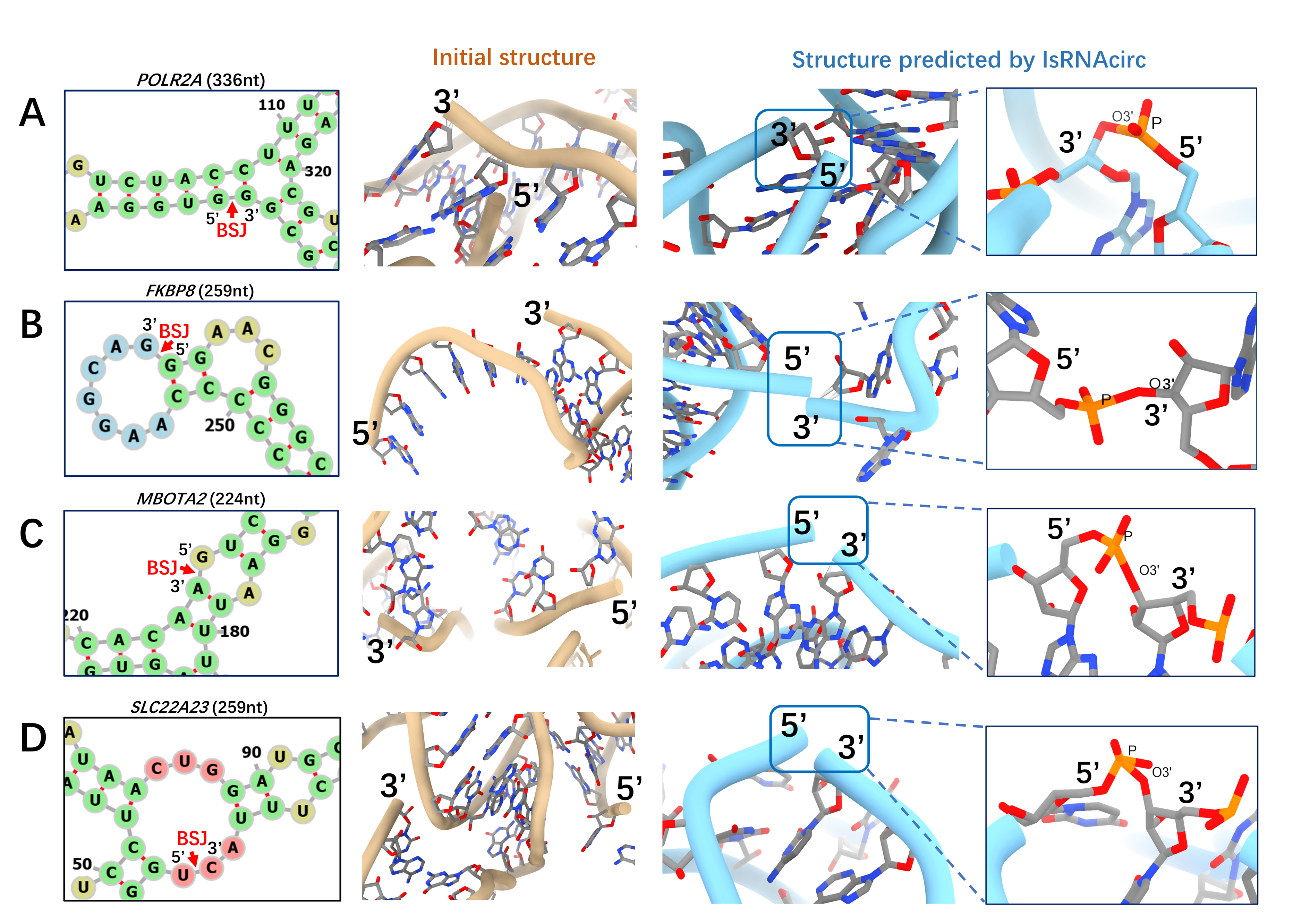

Supplement: S2 Fig — Illustrative examples depict the structural details of the 5’ and 3’ terminals of four distinct types of circular RNAs: (A) helical-circular, (B) hairpin-circular, (C) internal-circular, and (D) junction-circular RNA. From left to right, the secondary structure of the back-splice junction (BSJ) region, the 3D structure of the 5’ and 3’ terminals in the initial structure, and the circularized terminal nucleotides by IsRNAcirc. (TIF) [file pcbi.1012293.s002.tif]

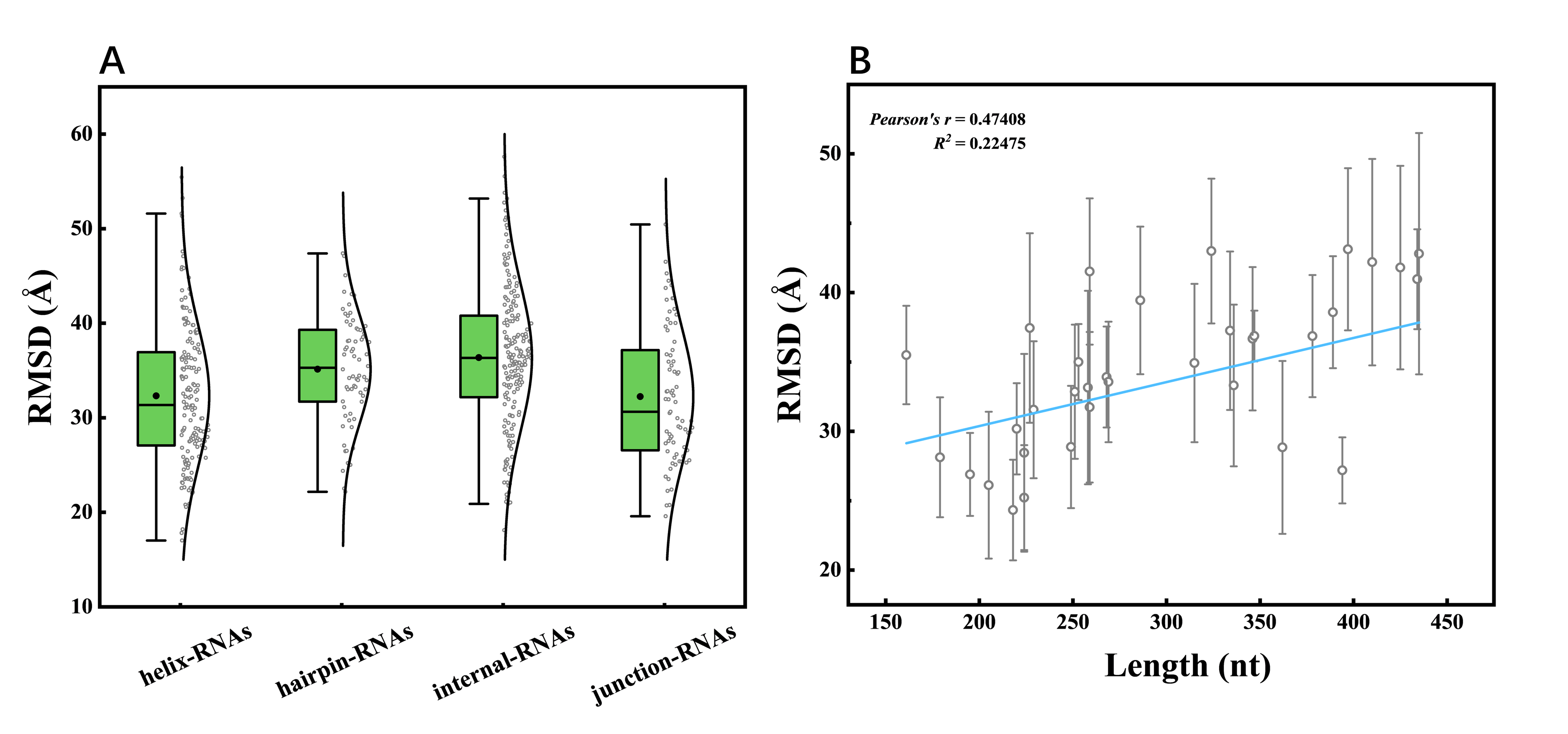

Supplement: S3 Fig — (A) Box plot of root-mean-square deviations (RMSDs) between the initial structure generated by RNAComposer and the predicted 3D structure by IsRNAcirc for four types of circular RNAs. The RMSD values were calculated by aligning the initial 3D structure with the 3D structure predicted by IsRNAcirc using PYMOL software. Boxes, interquartile range (IQR); center lines, median; black dots, mean; whiskers, values within 1.5 × IQR of the top and bottom quartiles. (B) Scatter plot of RMSD value as a function of the length of circular RNA. Values are mean ± s.d. (n = 12/15 independent RMSD values). (TIF) [file pcbi.1012293.s003.tif]

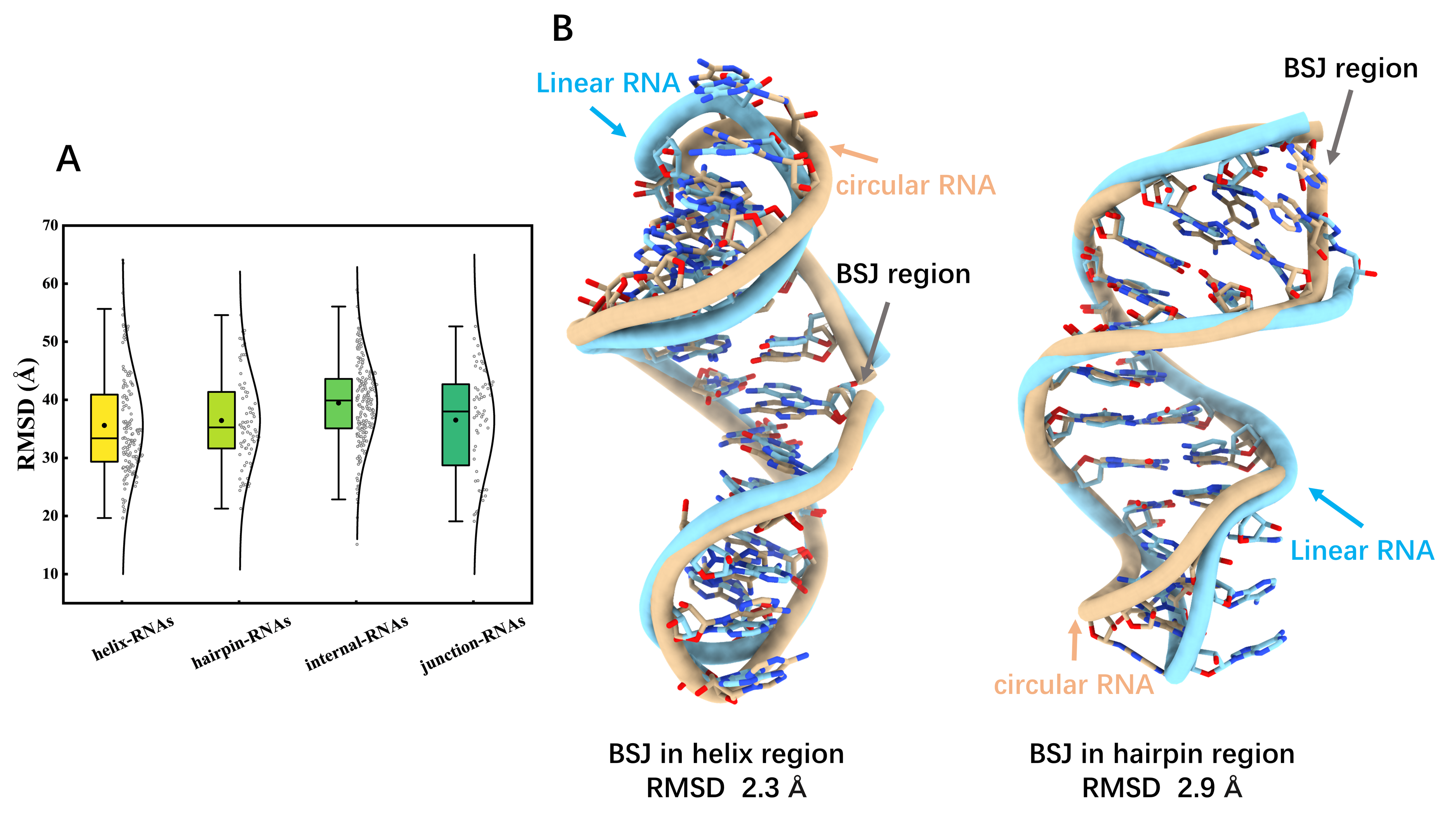

Supplement: S4 Fig — (A) Box plots of the pairwise RMSDs between the circular 3D models predicted by 3dRNA and their corresponding linear 3D structures generated by RNAComposer for all 34 tested circular RNAs. (B) Superposition of circular 3D structures predicted by IsRNAcirc and their corresponding linear 3D structure predicted by IsRNA2 for two constructed RNA molecules: helix-circular RNA (left, sequence: UCGUAAAAAACGAUCGAAAAACGA; 2D structure: “((((… ..))))(((… ..)))”) and hairpin-circular RNA (right, sequence: AACGAUCGUAAAAAACGAUCGAAA; 2D structure: “..(((((((… ..)))))))…”). (TIF) [file pcbi.1012293.s004.tif]

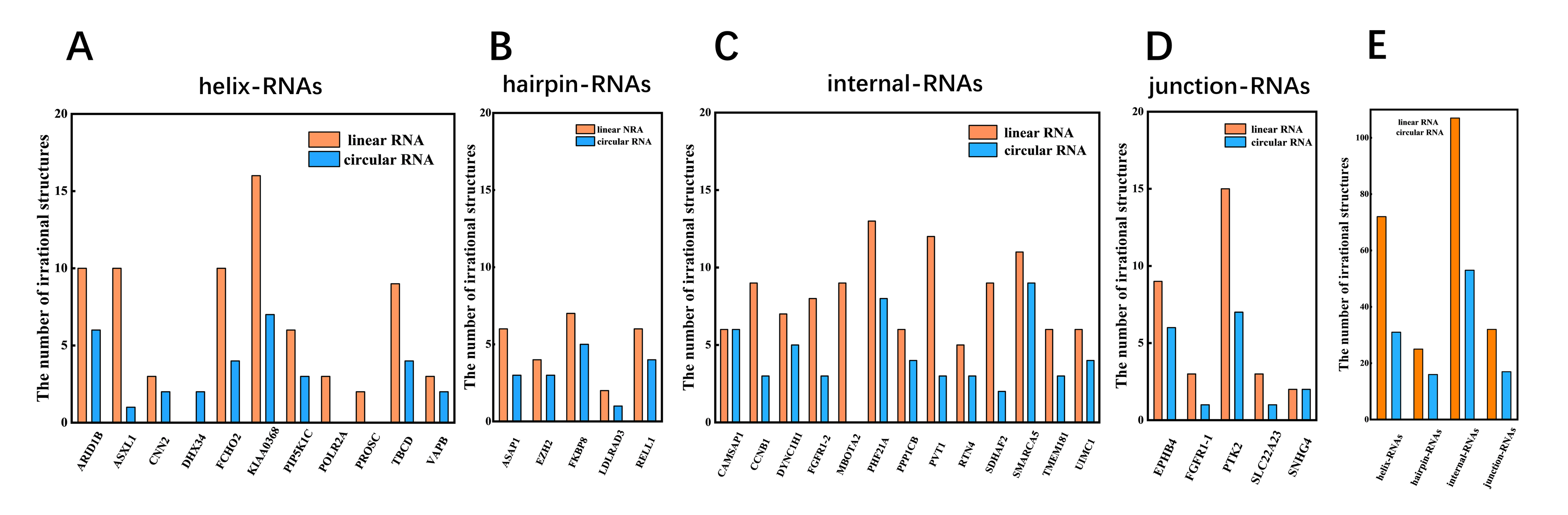

Supplement: S5 Fig — Detailed number of irrational structures in the 3D structure of each tested circular RNA: (A) helix-circular, (B) hairpin-circular, (C) internal-circular, and (D) junction-circular RNAs. (E) Total number of irrational structures for four types of circular RNAs. The initial structure of corresponding linear RNA and the predicted circular RNA structure by IsRNAcirc are shown in orange and blue bars, respectively. (TIF) [file pcbi.1012293.s005.tif]

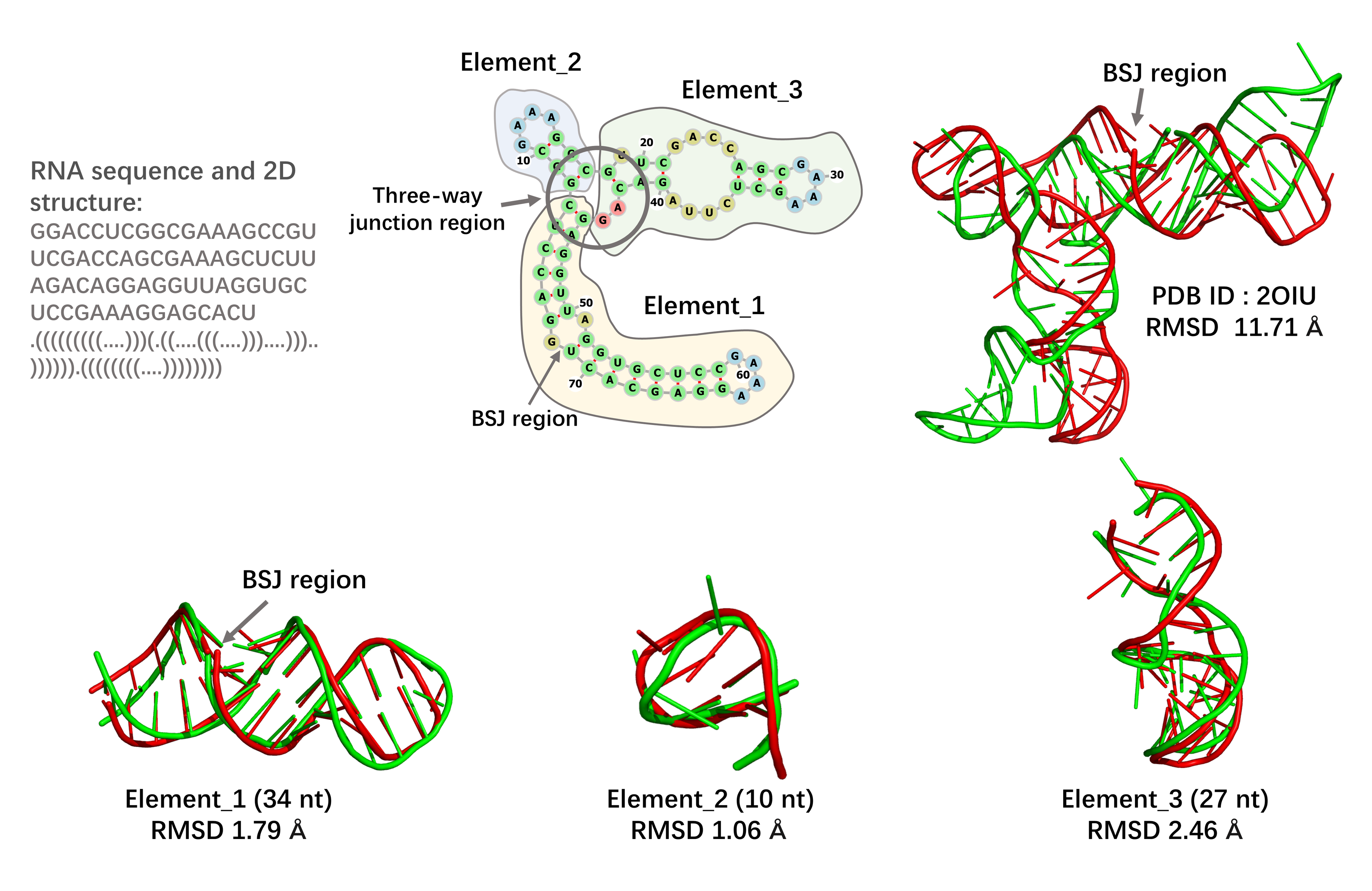

Supplement: S6 Fig — The sequence and native 2D structure extracted from the experimental structure were used as input. The experimental structure and the predicted 3D structure are colored in red and green, respectively. (TIF) [file pcbi.1012293.s006.tif]

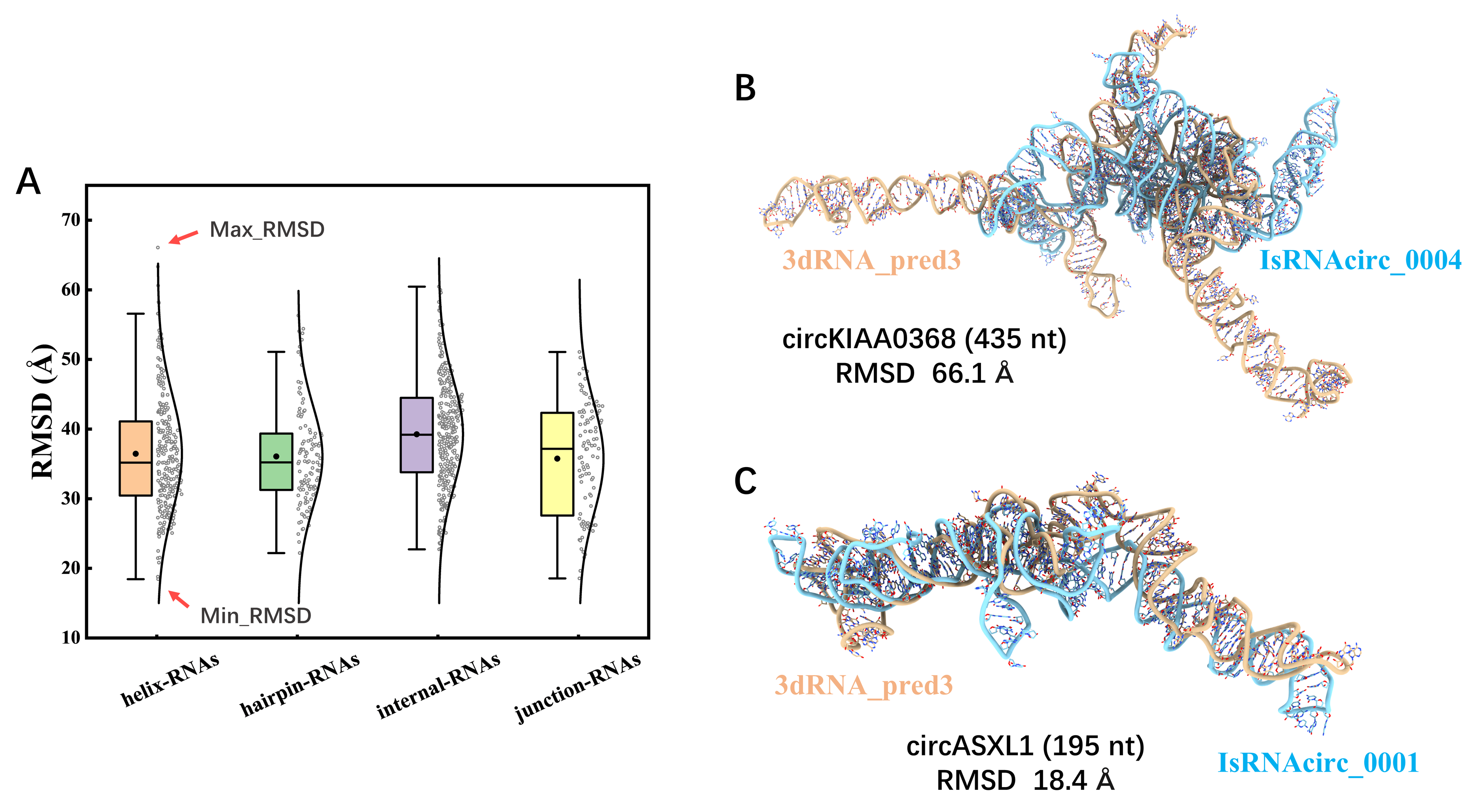

Supplement: S7 Fig — (A) Box plots of the pairwise RMSDs between the IsRNAcirc and 3dRNA predicted 3D structures for all 34 tested circular RNAs. (B) Superposition of the 3D structures of the circKIAA0368(435 nt) molecule predicted by IsRNAcirc and 3dRNA. (C) Superposition of the 3D structures of the circASXL1(195 nt) circular RNA predicted by IsRNAcirc and 3dRNA. (TIF) [file pcbi.1012293.s007.tif]

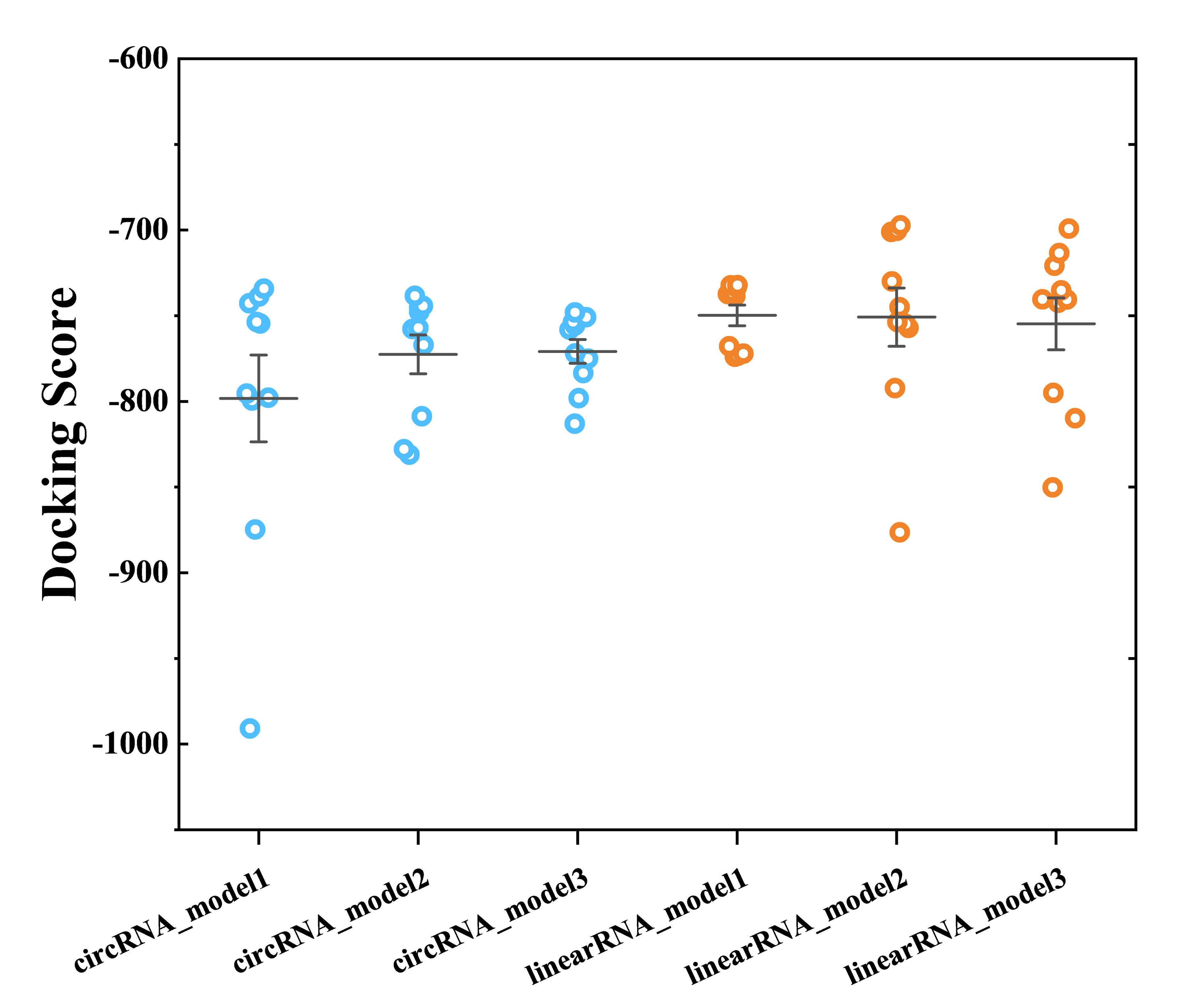

Supplement: S8 Fig — Three 3D models were prepared for the circPOLR2A molecule (predicted by IsRNAcirc) and its linear counterpart (predicted by IsRNA2), respectively. For each RNA 3D model, we used HDock to generate the top ten possible binding poses and recorded the associated docking scores. The mean±s.d. of the docking scores for each RNA 3D model was also shown. (TIF) [file pcbi.1012293.s008.tif]

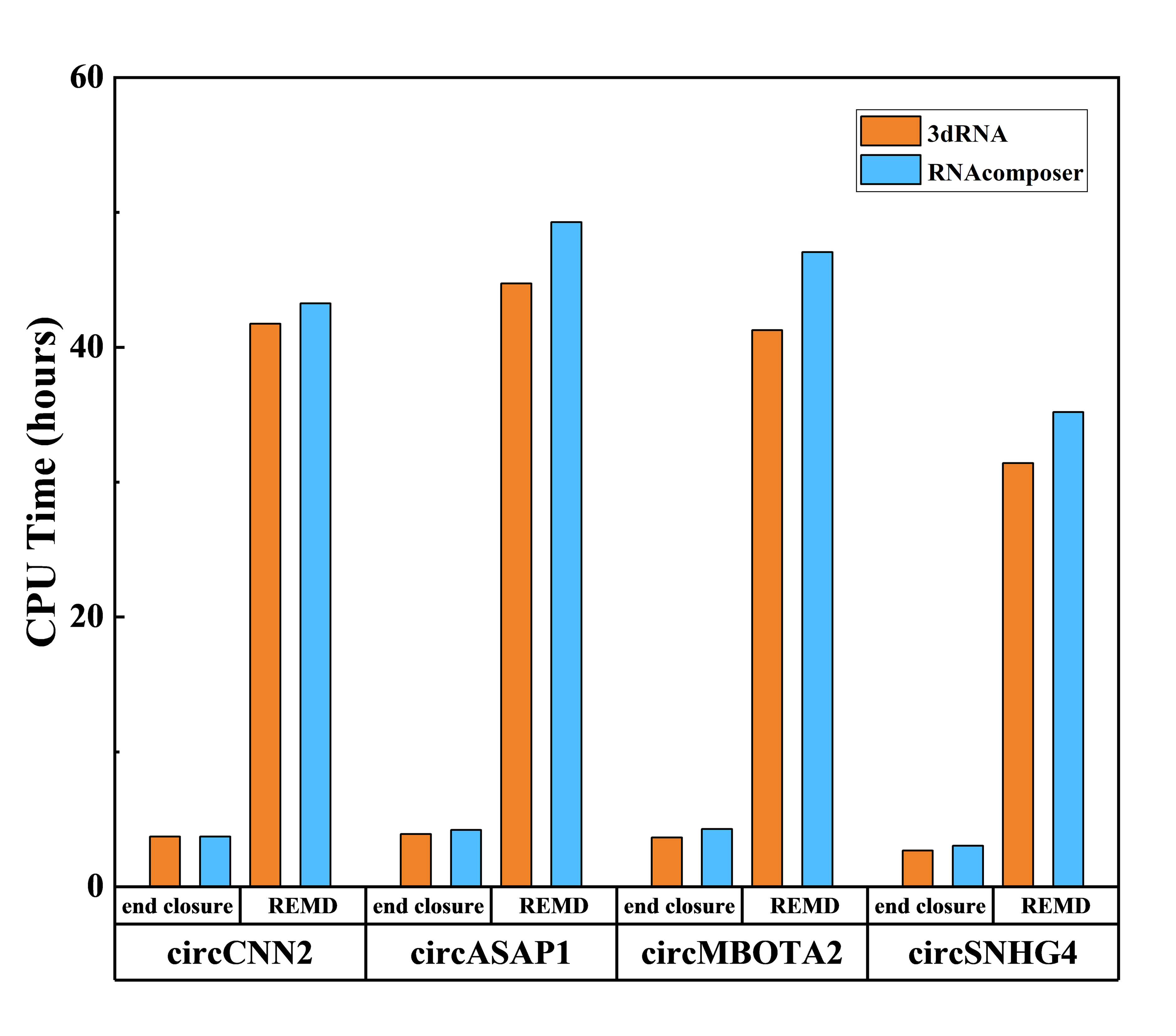

Supplement: S9 Fig — The runtimes for the end closure and structure prediction (REMD) stages were recorded separately. Predictions starting from the 3dRNA predicted circular 3D structure and the RNAComposer generated linear 3D structure were shown in orange and blue bars, respectively. (TIF) [file pcbi.1012293.s009.tif]
